# Olfactory and solitary chemosensory cells: two different chemosensory systems in the nasal cavity of the American alligator, *Alligator mississippiensis*

**DOI:** 10.1186/1471-2202-8-64

**Published:** 2007-08-03

**Authors:** Anne Hansen

**Affiliations:** 1 Department of Cell and Developmental Biology, Rocky Mountain Taste and Smell Center, University of Colorado Health Sciences Center, Anschutz Medical Campus, Mail Stop 8108, PO Box 6511, Aurora, CO 80045, USA

## Abstract

**Background:**

The nasal cavity of all vertebrates houses multiple chemosensors, either innervated by the Ist (olfactory) or the Vth (trigeminal) cranial nerve. Various types of receptor cells are present, either segregated in different compartments (e.g. in rodents) or mingled in one epithelium (e.g. fish). In addition, solitary chemosensory cells have been reported for several species. Alligators which seek their prey both above and under water have only one nasal compartment. Information about their olfactory epithelium is limited. Since alligators seem to detect both volatile and water-soluble odour cues, I tested whether different sensory cell types are present in the olfactory epithelium.

**Results:**

Electron microscopy and immunocytochemistry were used to examine the sensory epithelium of the nasal cavity of the American alligator. Almost the entire nasal cavity is lined with olfactory (sensory) epithelium. Two types of olfactory sensory neurons are present. Both types bear cilia as well as microvilli at their apical endings and express the typical markers for olfactory neurons. The density of these olfactory neurons varies along the nasal cavity. In addition, solitary chemosensory cells innervated by trigeminal nerve fibres, are intermingled with olfactory sensory neurons. Solitary chemosensory cells express components of the PLC-transduction cascade found in solitary chemosensory cells in rodents.

**Conclusion:**

The nasal cavity of the American alligator contains two different chemosensory systems incorporated in the same sensory epithelium: the olfactory system proper and solitary chemosensory cells. The olfactory system contains two morphological distinct types of ciliated olfactory receptor neurons.

## Background

The nasal cavity of all vertebrates houses multiple chemosensors. The olfactory and the vomeronasal receptors detect a variety of odours including food-related and social signals. In addition, chemically-sensitive free nerve endings of the trigeminal nerve and trigeminally innervated chemosensors that respond to irritants have been reported for some vertebrate species. The chemosensors are expressed in various cell types. In mammals, the olfactory system contains ciliated and microvillous olfactory receptor neurons (OSNs). In many mammals these neurons are segregated in two compartments: ciliated OSNs are housed in the main olfactory epithelium detecting chemicals related mostly to food and microvillous OSNs in the so-called vomeronasal organ (VNO) detecting mostly (but not limited to) social cues [1]. Fish olfactory epithelium also contains ciliated and microvillous OSNs [2], but here both cell types are intermingled in one olfactory epithelium since fish do not have a VNO. In mammals as well as in fish, both ciliated and microvillous OSNs utilize characteristic G-proteins in their transduction cascade: Gα_olf _is present in ciliated OSNs; Gα_i_, Gα_o _and/or Gα_q _are present in microvillous OSNs [3-7].

Chemoreception in semi-aquatic animals is interesting *per se *since both volatile and water-soluble compounds have to be detected in two different environments. Most semi-aquatic reptiles (turtles, tortoises, snakes) and amphibians possess a VNO lined with microvillous OSNs as well as the main olfactory chamber [8-11]. Turtles employ the main olfactory epithelium and the VNO to detect air-borne and/or water-soluble chemicals. However, this compartmentalization does not lead to a strict separation of volatile and soluble odorants. The VNO of semi-aquatic turtles responds to both volatile and non-volatile odorants [12]. Female tree frogs can detect aquatic sex pheromones from male frogs [13]. Axolotls (*Ambystoma mexicanum*) use their olfactory and vomeronasal systems equally to detect social cues from conspecifics [14]. The olfactory system of *Triturus pyrrhogaster*, changes morphologically and physiologically when the animals are kept either in a terrestrial or an aquatic environment [15,16].

Information about the nasal cavity of crocodilians is limited (for early macroscopic studies see [17]). This is not surprising given the difficulty of obtaining and handling the specimens. On the other hand, chemoreception in crocodilians (comprising the three families: Alligatoridae, Crocodylidae, and Gavialidae) is especially interesting since they hunt both in terrestrial and in aquatic surroundings. Crocodilians have only one olfactory chamber [18,19]. A VNO is absent in the adult animals. Thus, at first glance, crocodilians seem to have only one olfactory system comprising the main olfactory chamber and what type(s) of OSNs are present in the olfactory epithelium is unknown. Also, little is known about how crocodilians use their olfactory system. Neill [20] reported that the American alligator detects blood in the water. Behavioural and olfactometer experiments suggest that crocodilians detect both air-borne and water-soluble chemicals and use their olfactory system for hunting [18]. When above water, crocodilians enhance their ability to detect volatile odorants by gular pumping, a rhythmic movement of the floor of the pharynx [21,11,22]. If crocodilians do not have two different nasal compartments but detect both air-borne and water-soluble chemicals: do they have different types of olfactory neurons?

Also of interest is whether crocodilians possess trigeminally innervated solitary chemosensory cells (SCCs). These are specialized cells that have been described for anamniote vertebrates including hagfish [23], teleosts and amphibians [24-26]. SCCs, which are modified epithelial cells, represent a separate chemosensory system without a specific endorgan. These secondary receptor cells, i.e. lacking an axon, are scattered in different epithelia (e.g. skin, oropharyngeal cavity, nasal cavity, gills) and detect various chemical substances depending on the species investigated. These cells are scarce, and since they are scattered in the epithelium as single cells, no cell counts exist except for few fish species [27] and rats [28]. SCCs are also present in the nasal cavity and the respiratory tract of rodents where they detect irritants [28,29]. In rodents, SCCs express T2R "bitter-taste" receptors and α-gustducin, a G-protein involved in chemosensory transduction as well as phospholipase β2 (PLCβ2), a downstream component of the T2R-gustducin transduction cascade. Peptidergic fibres (containing Substance P/calcitonin gene-related peptide CGRP) of the trigeminal nerve form synaptic contacts with these cells [28]. Given the different origin and different innervation and transduction pathway of these receptor cells, it is obvious that SCCs represent a chemosensory system different from that of olfaction.

SCCs have also been reported for the oral and nasal cavity of cows [30] and the nasal epithelia of humans [31] but it is unclear whether the SCCs of mammals are related to the SCCs of anamniote vertebrates. The presence of similar SCCs in a reptile would support phylogenetic continuity rather than convergence between the anamniote and mammalian groups, suggesting that the SCCs are homologous throughout the vertebrate lineage.

The present study is the first to utilize electron microscopy and immunocytochemistry to examine the peripheral olfactory organ of a crocodilian, the American alligator *Alligator mississipiensis*. The study focussed on two questions: (1) What are the cell types in the nasal epithelium and does their ultrastructure and/or distribution give any clues regarding the differences between olfaction above or under water? Scanning and transmission electron microscopy was utilized to describe the cell types present. Furthermore, immunocytochemical tests with markers typically used for olfactory neurons were applied. (2) Do alligators possess solitary chemosensory cells (SCCs) thus suggesting that this chemosensory system is a conserved trait in the vertebrate lineage? The criteria for identifying SCCs were twofold: 1) morphology and 2) immunoreactivity to markers for SCCs. SCCs previously found in various vertebrates showed similarities in terms of cell shape and cell organelles. The cells are spindle-shaped with an apical ending that bears either one big villus or few smaller villi. At the surface of the epithelium, the apical ending is often constricted into a "neck". The cell body is filled with abundant vesicles of different shape and size [25,32,28]. A marker for the transduction pathway of SCCs is PLCβ2 [28,31,29]. Since SCCs are secondary receptor cells they are contacted by nerve fibres and these are immunoreactive for CGRP or Substance P.

## Results

### Overall Organization and Histology

**Figure 1:**
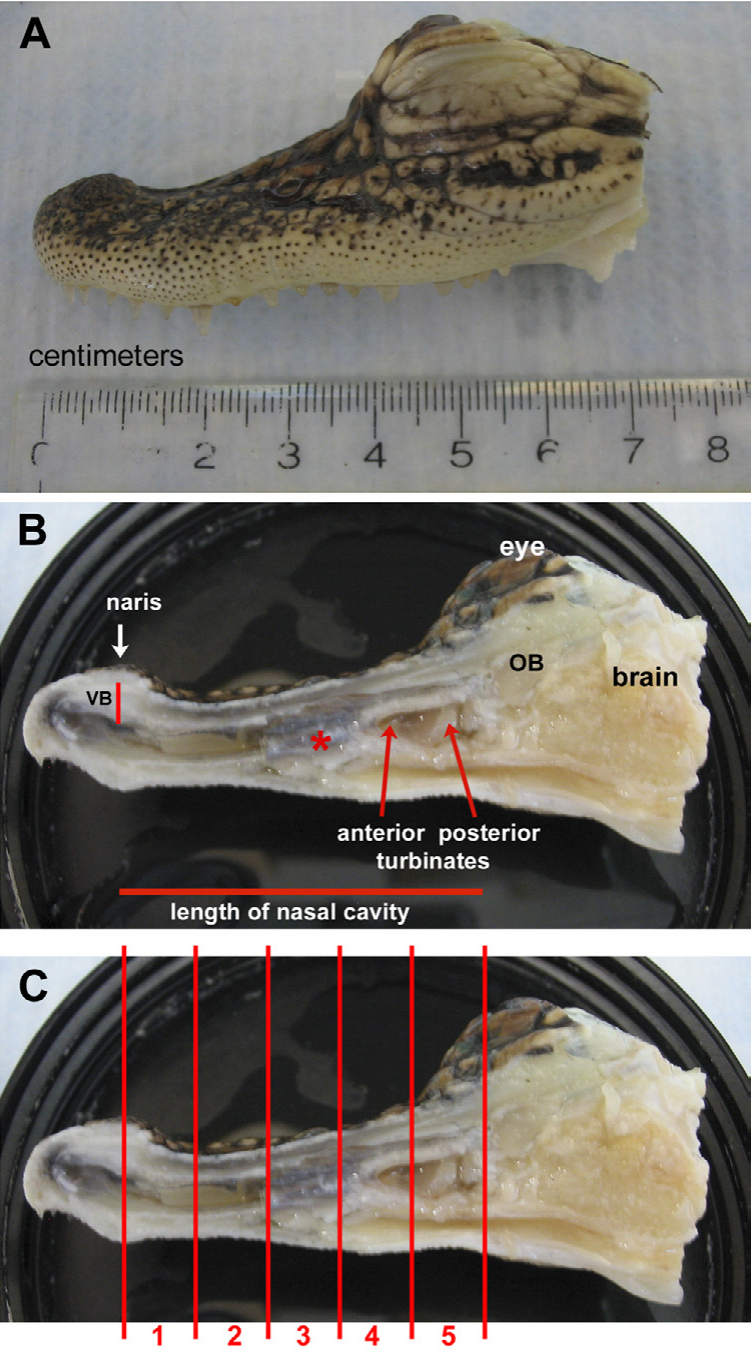
**Macroscopic view of split head of a juvenile alligator**. **A **Lateral view. **B **Right half of the head showing the incurrent naris and the length of the nasal cavity. * A piece of the septum occludes the nasal cavity. OB – olfactory bulb, VB – vestibule. **C **Same view as in **B **depicting the 5 zones that were examined at the light microscopic and the electron microscopic level.

**Figure 2:**
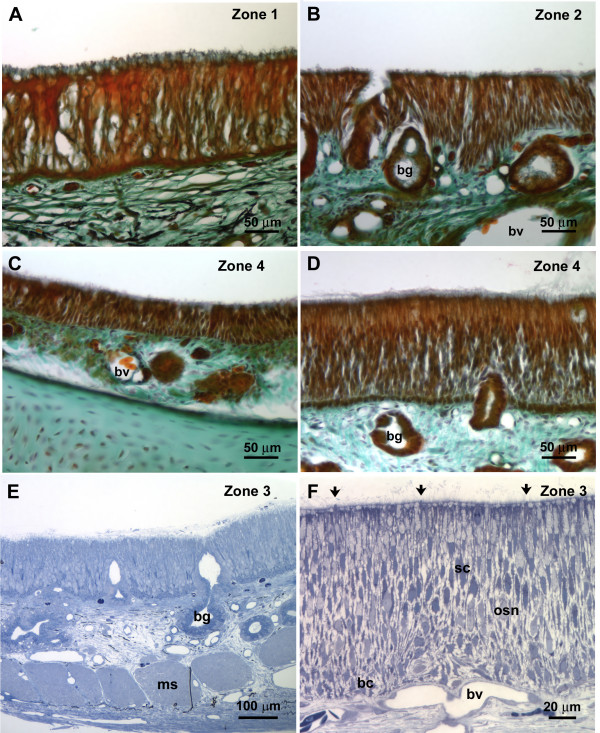
**A – D Histological staining nuclear red/light green/orange G**. Note that A – D are depicted at the same magnification and that all zones contain OSNs (based on results from semi- and ultrathin sections). **A **Olfactory epithelium in zone 1 close to the naris. **B **Ventrolateral olfactory epithelium in zone 2. The height of the epithelium is variable. Bowman glands (bg) are present. bv – blood vessel. **C **Olfactory epithelium in zone 4 (anterior turbinate). bv – blood vessel. **D **Olfactory epithelium in zone 4 (anterior turbinate) in a different region than shown in C. bg – Bowman gland. **E – F **Semithin (1 μm) sections stained with toluidin blue. Both **E **and **F **depict zone 3. Bowman glands are numerous (bg). Note in F how few OSNs are present (arrows). Sc shows a nucleus in the layer of nuclei of the supporting cells. osn shows the layer of nuclei of the olfactory neurons and bc the layer of basal cells. bg – Bowman gland, bv – blood vessel, ms – muscle.

**Figure 3:**
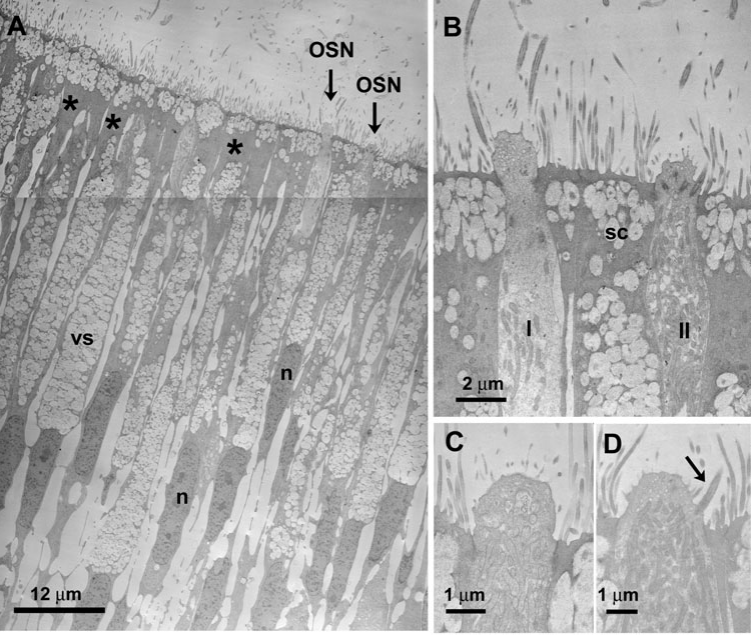
**TEM micrographs of alligator nasal epithelia**. **A **Low magnification of olfactory epithelium. Long slim supporting cells are filled with electron-lucent vesicles (vs). Note the area at the distal portion of the cell (asterisk) where vesicles are mostly absent. The surface of the supporting cells is covered with small microvilli. OSNs are rare (arrows). n – nucleus. **B **Higher magnification of the two different OSNs seen in **A**. One OSN (I) has few mitochondria. The cytoplasm is electron-lucent and contains microtubules. The other OSN (II) has abundant mitochondria and an electron-denser cytoplasm. Both cell types bear cilia and microvilli on their olfactory knobs. sc – supporting cell. **C **Apical ending of an OSN (type II) filled with mitochondria. The olfactory knob seems to bear only microvilli. However, serial sections reveal that cilia are present as seen in **D**. **D **The same type II OSN as in **C **showing microvilli and cilia.

**Figure 4:**
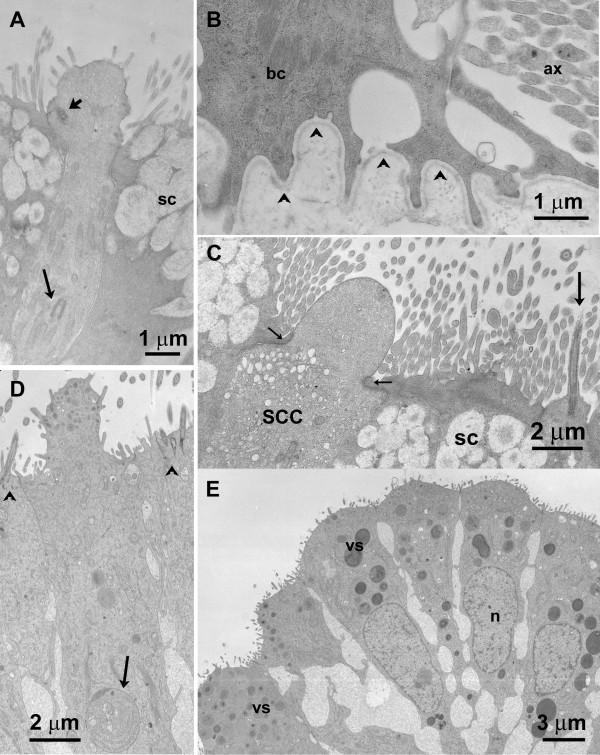
**TEM micrographs of alligator nasal epithelia**. **A **OSN (type I) with few mitochondria. The long arrow points to a centriole deep in the cytoplasm of the OSN. Short arrow – basal body of cilia. sc – supporting cell. **B **Basal portion of the olfactory epithelium. The basal lamina (arrowheads) follows the undulating shape of a basal cell (bc). Axons (ax) aggregate above the basal lamina into fila olfactoria that will penetrate the basal lamina in a different location. **C **Solitary chemosensory cell (SCC) in the olfactory epithelium. The cytoplasm is filled with small vesicles. The surrounding supporting cells (sc) constrict the apical ending of the SCC into a "neck" (small arrows). Large arrow – OSN. **D **Nonsensory epithelium in the most ventral part of the nasal cavity. Among ciliated nonsensory cells (arrowheads), a cell with small microvilli-like protrusions contains vesicles of various sizes (compare Fig. 5E). Debris-containing vesicles (arrow) suggest a phagocytotic function. **E **Another type of nonsensory epithelium with cells that bear no cilia but only small microvilli-like protrusions. The cytoplasm is also filled with vesicles of various sizes as seen in **D**, but cell debris never occurred within these cells. n – nucleus; vs – vesicles.

**Figure 5:**
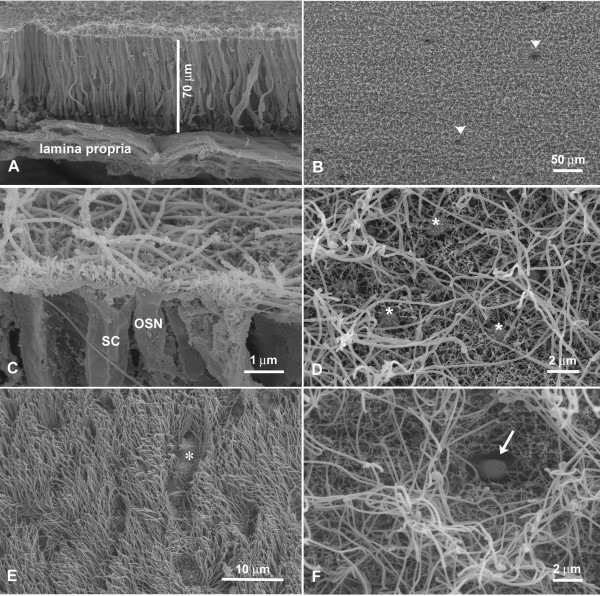
**SEM micrographs of alligator nasal epithelia**. **A **Fracture of olfactory epithelium in zone 5. With 70 μm this is one of the thinner regions of epithelium on the caudal turbinate. **B **Overview of nasal epithelium in zone 1 which TEM preparations proved to be olfactory. Arrowheads depict openings of Bowman glands. **C **Fracture showing the apical portion of olfactory epithelium in zone 4. The olfactory knob of an OSN bears cilia and microvilli whereas the apical surfaces of the supporting cells (SC) are covered by microvilli. **D **Overview of olfactory epithelium in zone 5 showing the "mixed" olfactory knobs bearing cilia and microvilli (*). Note that the cilia do not taper at their ends. The short microvilli of supporting cells are visible between the olfactory knobs. **E **Overview of nasal epithelium in zone 4. A dense population of ciliated nonsensory cells (compare Fig. 4D) covers the more ventrally located surface. Only occasionally, non-ciliated cells are interspersed (*). **F **Overview of olfactory epithelium in zone 5. Between the cilia of OSNs and supporting cells (distinguishable by their short microvilli), one thick dome-shaped apical ending indicates the presence of a SCC (arrow).

For a better understanding of the nasal cavity of alligators, cryosections of the nasal cavity were stained with Kernechtrot-Lichtgrün-Orange (KLO) (nuclear red-light green-orange G) and semithin sections were stained with toluidin blue [33]. To describe the olfactory epithelium macroscopically, the criteria commonly applied to olfactory epithelium were used. The receptor neurons are categorized according to their height and structure within the epithelium: tall cells with their nuclei in the lower portion of the epithelium and long slender dendrites are considered ciliated OSNs. Intermediate cells with their nuclei in the middle portion of the epithelium and short thick dendrites are considered microvillous OSNs (this latter cell type did not occur in the alligator epithelium) [34]. For convenience of description the nasal cavity was divided into 5 anterior-posterior zones, numbered 1 to 5 from anterior to posterior (Fig. 1C). The epithelium in each zone varies in thickness between 50 and 150 μm (Fig. 2). As seen in other vertebrates, the epithelium is pseudostratified with long, slender cells (Figs. 2E,F, 3A) that stretch from the basal lamina to the lumen of the nasal cavity. Bowman glands lie beneath the epithelium (Fig. 2B,D,E, 5B) and olfactory axons aggregate above and under the basal lamina (Fig. 4B). The occurrence of OSNs, however, is not limited to either the thin or the thick type of epithelium (see also *Immunocytochemistry*). As described before, almost the entire nasal cavity is lined with olfactory epithelium [19]. A few areas contain only nonsensory epithelium, mainly limited to the most ventral portion of the cavity and small ventral areas of the turbinates. These cells can be easily distinguished from the OSNs that have larger, more deeply placed nuclei. The vestibule (Fig. 1B), the duct leading from the naris opening down to the nasal cavity proper, is lined with short, flattened epithelial cells similar to those of the adjacent skin. OSNs are not present in the vestibule.

### Olfactory sensory neurons

The description of the ultrastructure of the OSNs follows the criteria commonly used for olfactory epithelium. As mentioned above, receptor cells are categorized macroscopically by their height within the epithelium. At the ultrastructural level, cell organelles are used to categorize OSNs, e.g. structural features of the cilia, presence or absence of rootlets, "checker-board" pattern of chromatin in nuclei, longitudinally arranged mitochondria, presence or absence of centrioles [35,36]. Electron micrographs (TEM) show that in the pseudostratified epithelium, the supporting cells are the most prominent (Fig. 3A). Their nuclei lie above the level of nuclei of OSNs. Abundant clear vesicles ca. 0.6 to 1.0 μm in diameter, fill the area above the nucleus and the most apical area leaving a small portion in between for mitochondria and electron-dense cytoplasm (Fig. 3A,B). Occasionally centrioles (usually two) are present in the upper portion of the cell which is devoid of vesicles (not shown). At the surface bordering the lumen, the width of the supporting cells ranges from 2.8 to 4.1 μm. This is only a rough estimate since these cells often envelop the OSNs. Consequently, at the ultrastructural level two supporting cell profiles, one on each side of an OSN, can be the same cell seen twice. The surface of the supporting cells is covered with numerous microvilli, about 4 to 5 μm long and 0.08 to 0.17 μm wide (Figs. 3B, 4C, 5A–C). OSNs are dispersed between the supporting cells (Figs. 3, 4A, 5C,D); the width of their apical dendrites (1 to 1.4 μm) is even smaller than the upper portion of the supporting cells. As seen in the scanning electron microscope (SEM), the pronounced olfactory knob bears about 6 to 7 cilia (10 to 15 μm long, approx. 0.3 μm wide) that do not taper at their end. In addition, the olfactory knobs bear several thin microvilli (about 1 μm long), and thus shorter and smaller than those of the supporting cells (Figs. 3B–D, 4A). Some TEM sections suggest the presence of OSNs with only microvilli. However, serial sections reveal that this is misleading (Figs. 3C, 4A), since the original plane of section may not include all of the cilia. All OSNs seen in serial ultrathin sections were so-called mixed receptor cells (bearing both cilia and microvilli) as described for birds by Graziadei [37]. Nevertheless, based on dendritic morphology, two different types of OSNs do exist. The distribution of these two types of OSNs within the nasal cavity has no obvious pattern. The olfactory knobs of both OSN types contain small light vesicles. Rootlets are absent from cilia of both cell types (Fig. 3B–D). Although both types bear cilia and microvilli on their olfactory knobs, their dendrites show different features. Type I OSNs are relatively electron-lucent and contain small vesicles (70–100 nm) and few microtubules. A few mitochondria are arranged longitudinally (Fig. 3B). Beneath the olfactory knob – often even deep in the dendrite – several centrioles are present (Fig. 4A), a feature that never occurred in the second OSN type. The cytoplasm of the type II OSN is electron-dense and filled with abundant mitochondria that sometimes reach into the olfactory knob. Their cytoplasm is electron-dense (Fig. 3B,C,D). Centrioles are lacking.

**Figure 6:**
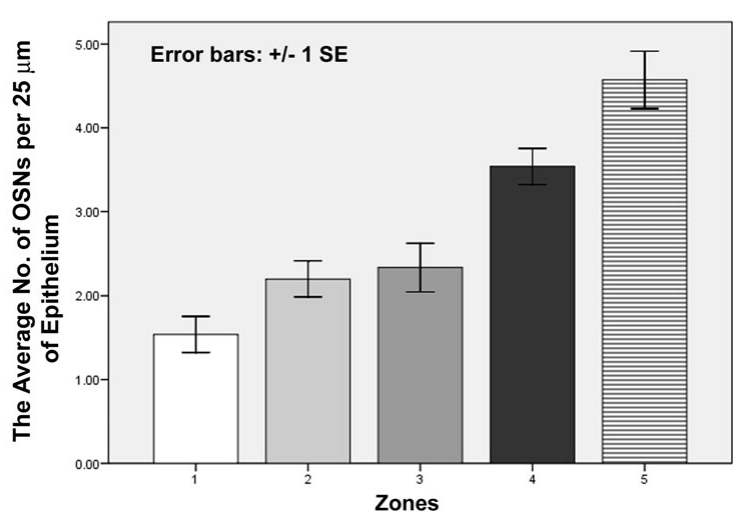
**Cell counting of sensory cells**. The average number of OSNs counted in the 5 different zones of the nasal cavity increases steadily from anterior towards the turbinates.

Compared to the olfactory epithelium of other groups of animals, the OSNs in the alligator's olfactory epithelium are rather scarce. In alligators, the density of OSNs varies in the different zones of the olfactory epithelium. The highest density occurs on the turbinates [4.6 (+/- 0.34 SE) apical knobs of OSNs per 25 μm length of epithelium, as assessed in ultrathin sections]. By way of comparison: goldfish or mouse olfactory epithelia contain approx. 8 – 10 OSNs per 25 μm length of olfactory epithelium in ultrathin sections (A.H., personal observation). Towards the rostral end of the nasal cavity of alligators, the density declines with the lowest numbers close to the vestibule [1.5 (+/- 0.22 SE) OSNs per 25 μm length of epithelium] (Fig. 6). ANOVA/Tukey tests of these cell countings revealed that zones 1, 2, and 3 were significantly different from zones 4 and 5. The difference between zone 4 and 5 was not significant (df between groups = 4, total = 87, F = 22.029, p < 0.05).

Beneath the layer of OSNs a layer of basal cells occurs. Basal cells rest on the basal lamina, their shape often following the curves of the basal lamina (Fig. 4B). Within the olfactory epithelium a few cells occur that contain cell debris enveloped in large vesicles (Fig. 4D). The nature of these presumably phagocytotic cells is unknown.

Strictly non-olfactory areas of epithelium are confined to the most ventral areas of the nasal cavity and small areas in the folding of the turbinates. These non-olfactory areas are mostly lined with columnar epithelial cells. They bear either tiny microvillous-like protrusions (Fig. 4E) or numerous cilia (Fig. 5E) which unlike cilia present on sensory neurons have pronounced rootlets extending into the cell (arrowheads Fig. 4D). Some of the microvilli-bearing cells are of mucous character (compare [19]) containing dark, electron-dense vesicles of various sizes. This part of the epithelium is loosely-packed and the wide interstitium is filled with dense extracellular matrix (Fig. 4E).

The antisera that worked in the alligator tissue corroborated the results obtained at the electron microscopic level.

**Figure 7:**
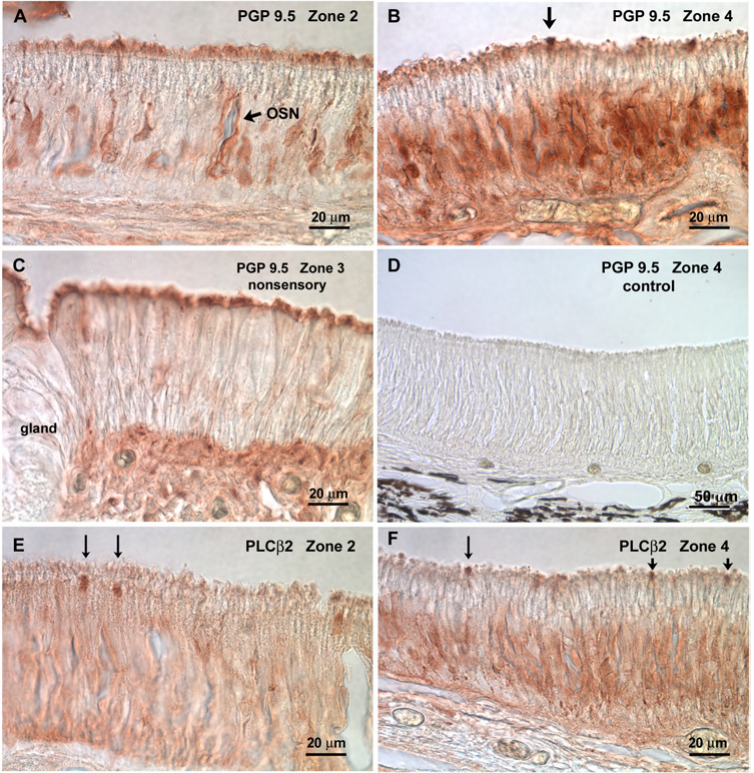
**Cryosections (16 μm) of various zones immunoreacted for PGP9.5 and PLCβ2**. **A **In zone 2 (anterior nasal cavity) few OSNs show PGP9.5-like immunoreactivity. **B **In zone 4 (anterior turbinate) considerably more OSNs are labelled with PGP9.5. Also, two larger apical endings (arrow) are darkly labelled (see also paragraph solitary chemosensory cells). **C **In the most ventral nonsensory portion of zone 3 no labelled cells occur. The apical fringe of nonsensory cilia is labelled non-specifically as is typical for nonsensory cilia. **D **A control section from zone 4 (anterior turbinate) where the primary antibody had been omitted shows no label at all. **E **PLCβ2 labels thick apical endings of few cells (arrows) in zone 2 (anterior nasal cavity) and also in **F **Zone 4 (anterior turbinate). Size and scarceness of the label indicates that these are SCCs.

**Figure 8:**
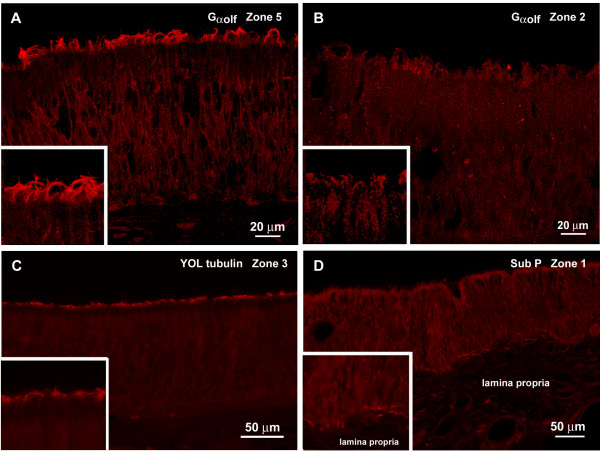
**Immunocytochemical experiments with fluorescent secondary antibodies**. The insets in A – C show higher magnifications of the apical and inset D the basal epithelia. **A **Gα_olf _labels a thick fringe of olfactory cilia in zone 5 (caudal turbinate). **B **In zone 2 (anterior nasal cavity) considerably fewer cilia are Gα_olf_-positive. **C **The YOL 1/34 antibody, a marker for microtubules, shows a fair amount of olfactory cilia in zone 3 (between the anterior turbinate and the anterior nasal cavity). **D **Substance P-positive fibers are present beneath the olfactory epithelium in zone 1 (most anterior nasal cavity). Some fibers reach into the epithelium where they may innervate SCCs.

(1) PGP9.5 showed labelled OSNs throughout the nasal cavity (Fig. 7A–B). The highest density of labelled cells was present in the turbinate areas (Fig. 7B), a lesser amount in the more anterior areas (Fig. 7A). Evidently, the height of the nasal epithelium did not correlate with sensory versus non sensory areas. PGP-positive OSNs occurred in areas with thick as well as in areas with thin epithelium. In nonsensory areas, PGP9.5 unspecific label was present in the nonsensory kinocilia (Fig. 7C). Occasionally, an apical ending that appeared to be dome-shaped showed PGP9.5-label (Fig. 7B). These cells may be SCCs (see below).

(2) Gα_olf_, a marker for the canonical transduction cascade in olfaction, was present in the apical endings of OSNs (Fig. 8A–B). The label occurred throughout the nasal cavity proving the presence of OSNs in wide areas of the nasal epithelium. The degree of label, however, varied from area to area with the strongest label in the turbinate areas (Fig. 8A) and the weakest label in the anterior areas of the cavity (Fig. 8B) corresponding to PGP 9.5 results showing fewer OSNs in the anterior areas (Fig. 7A). Also as seen in PGP 9.5 experiments, Gα_olf _occurred both in thick and in thin epithelia.

(3) YOL 1/34 tubulin (a marker for microtubules) immunoreaction is present in cilia (Fig. 8C) and in fibres coursing below the olfactory epithelium but no positive fibres are seen beneath the nonsensory areas.

### Solitary chemosensory cells

Occasionally, cells with the morphology of solitary chemosensory cells (SCCs) occur in the olfactory epithelium. These cells are scarce but stand out in SEM and TEM preparations (Figs. 4C, 5F). Their apical ending is dome-shaped. Supporting cells seem to indent into the upper part of the cell resulting in a "neck" of the presumed SCCs (Fig. 4C). The dome is filled with dense cytoplasm and few tiny vesicles (approx. 0.02 – 0.05 μm). The area below the "neck" on the other hand contains abundant slightly larger vesicles (approx. 0.07 – 0.2 μm).

Immunocytochemical results (synapses in the lower portion of the epithelium, PLCβ2 in apical endings) corroborate the notion that these cells are, indeed, SCCs.

(1) PLCβ2, a marker for the transduction pathway of SCCs and some OSNs [28,38], labels few apical endings which at the light microscopic level appear to be dome-shaped (Fig. 7E–F).

(2) Substance P labels nerve fibres under the nasal epithelium. Some of the fibres reach into the olfactory and into the nonsensory epithelium (Fig. 8D). Other Substance P-positive fibres are present in the close vicinity of the glands.

## Discussion

The present study is the first to utilize electron-microscopic and immunocytological methods on the olfactory epithelium of a crocodilian species. The findings show that almost the entire nasal cavity is lined with olfactory (= sensory) epithelium, but the density of the olfactory receptor neurons (OSNs) varies along the nasal cavity. Two different types of ciliated OSN exist that vary in cell organelles and electron-density. The nasal cavity contains not only olfactory sensory neurons (OSNs), but also receptor cells of a second chemosensory system: the solitary chemosensory cells (SCCs) which connect to peripheral fibres of the trigeminal nerve.

Early studies on crocodilians focused on the macroscopic morphology of the head and described the nasal cavity only briefly and mostly in terms of head morphology (for a more complete literature list on head morphology see [17]. Saint Girons [19] investigated the nasal cavities of *Crocodylus niloticus *and *Caiman crocodilus *with histological methods.

### Olfactory sensory neurons

The present study of the American alligator confirms Saint Girons' [19] findings insofar as almost the entire nasal cavity is lined with olfactory (= sensory) epithelium. However, the density of OSNs is heterogeneous along the nasal cavity of the American alligator. The highest numbers of OSNs occur in the zones of the two turbinates and the lowest numbers close to the naris. Saint Girons' [19] cell countings in histologically stained sections rendered a proportion of 61% OSNs for *Crocodylus *and 59% of OSNs for *Caiman*. These results are very different from what I counted in electron microscopic experiments: In the alligator, the proportion of OSNs versus supporting cells is reversed compared to Saint Girons' countings [19]: the olfactory epithelium contains many more supporting cells than OSNs – even in the turbinate zones where the OSNs have the highest density (Fig. 6). The difficulties in counting supporting cells cannot account for this considerable difference. This could be species-dependent but it is more likely due to the very different techniques. Saint Girons [19] counted nuclei in histologically stained thick sections which is tedious but not very reliable due to the poor resolution and thus difficulties in distinguishing cell types, whereas in the present study cells were counted at the ultrastructural level.

Since alligators hunt above and under water and given the fact that they possess only one nasal compartment the question arises how are volatile and/or water-soluble chemicals detected? A study on the semi-aquatic newt *Triturus pyrrhogaster *reported a change of morphology and physiological properties of the OSNs depending on the environment. In a terrestrial environment the cilia of the OSNs grew longer than when they were in aquatic surroundings. Slow potentials evoked in the olfactory epithelium changed from responding to odorous fluids in an aqueous medium to responding to odorous vapours in a terrestrial environment [16]. Interestingly, two types of OSNs are present in the olfactory epithelium of the American alligator. Both types bear cilia that do not taper at their ends as seen in rodents. Other than that, the two types of ciliated OSNs show different morphological features that are commonly used to describe OSNs (number of mitochondria, presence/absence of centrioles, electron density of cytoplasm [35]) so that type I and type II OSNs are easily distinguished at the ultrastructural level. This has not been reported for birds (the second group of the Sauropsida) or other reptiles. What the two different types of OSNs mean in terms of function is yet unknown, but it is tempting to speculate that one type of OSN may be utilized above water for air-borne stimuli while the other is used under water. The results described in this study were collected from juvenile specimens. It remains to be shown whether the findings also apply to fully mature alligators.

Immunocytochemical experiments were hampered by the fact that most of the antisera made for rodents do not work in alligators. Nevertheless, it was possible to show that, as seen in fish and mammals, the OSNs in alligators express Gα_olf_, the most common G-protein subunit in olfactory transduction [3,6,7]. The neuronal marker PGP9.5 confirmed the presence of OSNs in various areas. This was especially helpful since it corroborated on a larger scale what ultrastructural experiments had shown: (a) that the density of OSNs varies from the anterior end of the snout to the turbinates, and (b) that the height of the epithelium is not correlated to the presence/absence of OSNs. They occur in thicker as well as in thinner areas of epithelium. These two features were also evident in experiments with the YOL 1/34 antibody, a marker for tubulin in cilia, and Gα_olf_.

### Solitary chemosensory cells

Another interesting feature of the alligator's nasal epithelium is the presence of SCCs. These chemoreceptor cells had been known for fish and amphibians [24,25]. They occur in the skin of the body of fish, in the oral cavity and on the gills [26,39]. In the alligator, SCCs are integrated into the olfactory epithelium, a feature that up to now has been reported only for the goldfish olfactory epithelium [40]. With some exceptions, their function is not well understood. Recently, SCCs were also described for rodents [28]. In this case, the SCCs are located in the respiratory epithelium of the nasal cavity. In rodents, the SCCs express components of the T2R transduction cascade including gustducin and PLCβ2. Electrophysiological and behavioural experiments led to the conclusion that SCCs in rodents serve as "sentinels" in the anterior nasal cavity guarding against potentially harmful or irritant chemicals. SCCs also occur in guinea pigs [41] and cows [30]. In alligators, dome-shaped apical endings were labelled with PLCβ2 as are nasal SCCs in rodents. In addition, Substance P-positive fibres were present in and under the nasal epithelium of the alligator indicating innervation of the SCCs by trigeminal nerve fibres as seen in rodents. The presence of SCCs in alligators as well as in hagfish [23], elasmobranchs, teleost fish [25], amphibians [24,42], rodents [28], large mammals [30], and humans [31] support the notion that SCCs are an evolutionarily conserved trait having evolved early in vertebrate phylogeny.

## Conclusion

Two different chemosensory systems are incorporated in the olfactory epithelium: olfactory sensory neurons proper and solitary chemosensory cells comprised of secondary sensory cells innervated by the trigeminal nerve. Almost the entire nasal cavity of the American alligator is lined with olfactory (sensory) epithelium. Two morphologically distinct types of ciliated OSNs are present. Although both types bear cilia as well as microvilli on their olfactory knobs, they differ in electron density and cell organelles. The density of OSNs varies with the lowest numbers close to the nares and the highest numbers on the turbinates. OSNs as well as SCCs in alligators express members of the transduction cascade seen in rodents. The occurrence of SCCs in the alligator suggests phylogenetic continuity of this cell type throughout the vertebrates.

## Methods

### Animals

Three juvenile alligator heads (Fig. 1A) fixed in 4% paraformaldehyde (kindly provided by Dres. Catherine Carr and Daphne Soares, University of Maryland) were split at the midline by means of a fretsaw (Fig. 1B). The complete nasal sacs were dissected out and divided into 5 zones for further processing (Fig. 1C). Two animals were used for transmission electron microscopy and immunocytochemistry, the third animal for scanning electron microscopy and immunocytochemistry.

### Light microscopy

For an overview at the light microscopic level, cryosections (16 μm) were treated with the histological staining Kernechtrot-Lichtgrün-Orange (KLO) (0.1% nuclear red – C.I. 60760, Merck, Darmstadt – in 5% aluminium sulphate followed by 0.2% light green – C.I. 42095, Allied Chemical Corp., New York, 1% orange G – C.I. 16230, Allied Chemical Corp., New York – and 0.5% phosphotungstic acid in 1% acidic acid) [33]. Briefly, the slides were washed in dH_2_O, immersed in a mixture of nuclear red in aluminium sulphate for 10 min, washed in dH_2_O, and immersed in a mixture of light green and orange G in phosphotungstic acid for 1 – 2 min. After the staining process, the slides were dehydrated in ethanol and xylene. Epon-Araldite-embedded pieces of tissue were cut at 1 μm and stained with 1% toluidin blue (J. T. Baker Chemical Co., Phillipsburg).

### Scanning electron microscopy (SEM)

Pieces of tissue of all 5 zones of the nasal cavity were postfixed in 4% glutaraldehyde in 0.05 M phosphate buffer (pH 7.2). After rinsing in phosphate buffer, the specimens were dehydrated in a graded series of acetone and isoamyl acetate, critical-point-dried in CO_2_, coated with gold, and examined with a LEO 1525 scanning electron microscope (Carl Zeiss, Jena, Germany).

### Transmission electron microscopy (TEM)

Pieces of tissue of all 5 zones of the nasal cavity were postfixed in 4% glutaraldehyde in 0.05 M phosphate buffer (pH 7.2) for several hours. After rinsing in phosphate buffer, the tissue samples were postfixed with 1% osmium tetroxide for 2 h. The fixed specimens were dehydrated in a graded series of ethanol and acetone and embedded in Epon-Araldite (Electron Microscopy Sciences, Hatfield, PA). Ultrathin sections (silver to gold) were stained with uranyl acetate and lead citrate and examined with a FEI Tecnai G^2 ^electron microscope (Philips, Eindhoven, Netherlands).

### Cell counting

Epithelia of all 5 zones were photographed in the transmission electron microscope and printed at a magnification of 4000×, the lowest magnification where it was possible to unequivocally distinguish cell types. Photos of each zone were taken in a single session to ensure that the epithelia were captured only once. Thus, double counting of cells was prevented. On these photos, the apical portion of the epithelium was divided into sections (87 in total) each representing 25 μm of actual tissue. Only OSNs that reached the apical surface were counted. The investigator was blinded as to location of the sample at the time each was counted. Statistical analysis (ANOVA, Tukey test) was done with the program SPSS 15.0 (SPSS Inc., Chicago).

### Immunocytochemistry

Antisera directed against various markers for OSNs and SCCs were used to test their effectiveness in alligator tissue. These experiments were hindered by the fact that most antisera directed against mammalian proteins did not work in alligators (e.g. NCAM, gustducin, CGRP, Hu-C, calbindin, calretinin, RMRF, OMP, Gα_o_). Consequently, it is not possible to tell whether these proteins are present or not by this method. A further difficulty was the high autofluorescence of the tissue. All antisera were used with fluorescence-coupled and biotinylated secondary antibodies. Best results were obtained with antigen retrieval, extensive blocking of endogenous peroxidase and biotinylated secondary antibodies. 16 μm cryosections were processed according to standard methods. Briefly, the sections were subjected to either acidic (30 min in 10 mM citric acid, pH 3.0, at 37 degrees C) or basic (30 min in 0.05 M Tris buffer, pH 8.0, at 80 degrees C) antigen retrieval. After three washes in phosphate-buffered saline (PBS; pH 7.2), the sections were left in blocking solution [1% bovine serum albumin, 5% normal horse serum (NHS), 0.3% Triton X-100] for 2 – 3 hours. Incubation with the primary antisera diluted in blocking solution containing 2% NHS lasted overnight to 3 days. After three washes in PBS, fluorescence-coupled secondary antibodies were used to visualize the antibody labelling. As the autofluorescence of the tissue was usually very high, more immunocytochemical experiments were carried out with biotinylated secondary antibodies. In that case, endogenous peroxidase was quenched with 0.3% H_2_O_2 _for 30 min. After 3 × 15 min washes in PBS, the blocking solution was applied as described above. Then the slides were incubated in avidin (Blocking Kit, Vector Laboratories Inc., Burlingame, CA) for 15 min, rinsed briefly and incubated in biotin (Blocking Kit, Vector Laboratories Inc., Burlingame, CA) for 15 min. The slides were left in the primary antisera overnight and reacted with the ABC/DAB method according to standard protocols. As a control, various steps (primary antibody, secondary antibody, ABC, DAB) were omitted. The antisera that immunolabeled alligator tissue were: rabbit Protein Gene Product 9.5 (PGP9.5 = Ubiquitin C-terminal Hydrolase, dilution 1:200 – 1:1000, Biogenesis, Kingston, NH), rabbit Gα_olf _(dilution 1:1000, St. Cruz Biotechnologies, Santa Cruz, CA), rabbit PLCβ2 (dilution 1:100, St. Cruz, Santa Cruz Biotechnologies, Santa Cruz, CA), rat Substance P (dilution 1:100, Accurate Chemical and Scientific Corp., Westbury, NY), rat YOL 1/34 tubulin (dilution 1:100 – 1:200, Abcam, Inc., Cambridge, MA) (Table 1).

**Table 1:** Antisera used for immunocytochemistry

	**Company**	**Cat. No**.	**Lot No**.	**host animal**	**Dilution**
Gαolf	Santa Cruz	sc383	K189/L0602	rabbit	1:1000
PGP9.5	Biogenesis	7863-0504	24101201	rabbit	1:200–1:1000
PLCβ2	Santa Cruz	sc206	A1204	rabbit	1:100
Sub P	Accurate	YMC1021	E9381	rat	1:100
YOL 1/34	Abcam	AB6161	165477	rat	1:100–1:200

## Authors' contributions

AH designed the study, carried out all experiments and prepared the manuscript.

## References

[B1] Farbman AI (1992). Cell Biology of Olfaction.

[B2] Bannister LH (1965). The fine structure of the olfactory surface of teleostean fishes. Quart J Microsc Sci.

[B3] Jones DT, Reed RR (1989). Golf: an olfactory neuron specific-G protein involved in odorant signal transduction. Science.

[B4] Wekesa KS, Anholt RRH (1999). Differential expression of G proteins in the mouse olfactory system. Brain Res.

[B5] Wekesa KS, Miller S, Napier A (2003). Involvement of Gq/11 in signal transduction in the mammalian vomeronasal organ. J Exp Biol.

[B6] Hansen A, Rolen SH, Anderson KT, Morita Y, Caprio J, Finger TE (2003). Correlation between olfactory receptor cell type and function in the channel catfish. J Neurosci.

[B7] Hansen A, Anderson KT, Finger TE (2004). Differential distribution of olfactory receptor neurons in goldfish: structural and molecular correlates. J Comp Neurol.

[B8] Hatanaka T, Matsuzaki O, Shibuya T (1982). Fine structure of vomeronasal receptor cells in the Reeve's turtle, Geoclemys reevesii. Zool Mag.

[B9] Hansen A, Reiss JO, Gentry CL, Burd GD (1998). Ultrastructure of the olfactory organ in the clawed frog, Xenopus laevis, during larval development and metamorphosis. J Comp Neurol.

[B10] Reiss JO, Eisthen HL (2007). Chemical Senses: comparative anatomy and physiology in amphibians. Senses on the Threshold, J G H Thewissen, S Nummela (eds ), University of California Press, Berkeley.

[B11] Halpern M, Martinez-Marcos A (2003). Structure and function of the vomeronasal system: an update. Progr Neurobiol.

[B12] Hatanaka T, Matsuzaki O (1993). Odor responses of the vomeronasal system in Reeve's turtle, Geoclemys reevesii. Brain, Behav Evol.

[B13] Wabnitz PA, Bowie JH, Tyler MJ, Wallace JC, Smith BP (1999). Aquatic sex pheromone from a male tree frog. Nature.

[B14] Park D, McGuire JM, Majchrzak JM, Ziobro JM, Eisthen HL (2004). Discrimination of conspecific sex and reproductive condition using chemical cues in axolotls (Ambystoma mexicanum). J Comp Physiol A.

[B15] Matthes E (1927). Der Einfluss des Mediumwechsels auf das Geruchsvermögen von Triton. Z vergl Physiol.

[B16] Shibuya T, Takagi SF (1963). Electrical response and growth of olfactory cilia of the olfactory epithelium of the newt in water and on land. J Gen Physiol.

[B17] Bertau M (1935). Zur Entwicklungsgeschichte des Geruchsorgans der Krokodile. Z Anat EntwGesch.

[B18] Weldon PJ, Swenson DJ, Olson JK, Brinkmeier WG (1990). The American alligator detects food chemicals in aquatic and terrestrial environments. Ethology.

[B19] Saint Girons H (1976). Données histologiques sur les fosses nasales et leurs annexes chez Crocodylus niloticus Laurenti et Caiman crocodilus (Linnaeus) (Reptilia, Crocodylidae). Zoomorphologie.

[B20] Neill WT (1971). The Last of the Ruling  Reptiles: Alligators, Crocodiles, and Their Kin.

[B21] Gans C, Clark B (1976). Studies on ventilation of Caiman crocodilus (Crocodilia: Reptilia). Respir Physiol.

[B22] Putterill JF, Soley JT (2006). Morphology of the gular valve of the Nile crocodile, Crocodylus niloticus (Laurenti, 1768). J Morphol.

[B23] Braun CB, Northcutt RG, Jørgenson JM, Lomholt JP, Weber RE and Malte H (1998). Cutaneous exteroreceptors and their innervation in hagfishes. The Biology of Hagfishes.

[B24] Morrill AD (1895). The pectoral appendages of Prionotus and their innervation. J Morphol.

[B25] Whitear M (1965). Presumed sensory cells in fish epidermis. Nature.

[B26] Kotrschal K, Krautgartner WD, Hansen A (1997). Ontogeny of the solitary chemosensory cells in the zebrafish, Danio rerio. Chem Senses.

[B27] Kotrschal K (1992). Quantitative scanning electron microscopy of solitary chemoreceptor cells in cyprinids and other teleosts. Environ Biol Fishes.

[B28] Finger TE, Böttger B, Hansen A, Anderson KT, Alimohammadi H, Silver WL (2003). Solitary chemoreceptor cells in the nasal cavity serve as sentinels of respiration. PNAS.

[B29] Merigo F, Benati D, Tizzano M, Osculati F, Sbarbati A (2005). α-Gustducin immunoreactivity in the airways. Cell Tissue Res.

[B30] Tizzano M, Merigo F, Sbarbati A (2006). Evidence of solitary chemosensory cells in a large mammal: the diffuse chemosensory system in Bos taurus airways. J Anat.

[B31] Hansen A (2005). Unconventional receptor cells in the nasal cavity of humans and rodents. Soc Neurosci Abstr.

[B32] Hansen A, Kapoor BG and Reutter K (2005). The system of solitary chemosensory cells. Fish Chemosenses.

[B33] Romeis B, Böck P (1989). Mikroskopische Technik.

[B34] Morita Y, Finger TE (1998). Differential projections of ciliated and microvillous olfactory receptor cells in the catfish, Ictalurus punctatus. J Comp Neurol.

[B35] Hansen A, Zielinski BS (2005). Diversity in the olfactory epithelium of bony fishes: development, lamellar arrangement, sensory neuron cell types and transduction components. J Neurocytol.

[B36] Graziadei PPC, Monti-Graziadei GA (1976). Olfactory epithelium of Necturus maculosus and Ambystoma tigrinum. J Neurocytol.

[B37] Graziadei PC, Bannister LH (1967). Some observations on the fine structure of the olfactory epithelium in the domestic duck. Z Zellforsch.

[B38] Lin W, Arellano J, Slotnick B, Restrepo D (2004). Odors detected by mice deficient in cyclic nucleotide-gated channel subunit A2 stimulate the main olfactory system. J Neurosci.

[B39] Finger TE (1997). Evolution of taste and solitary chemoreceptor cell systems. Brain Behav Evol.

[B40] Hansen A, Zippel HP, Sorensen PW, Caprio J (1999). Ultrastructure of the olfactory epithelium in intact, axotomized, and bulbectomized goldfish, Carassius auratus. Microsc Res Techn.

[B41] Taylor-Clark TE, Kollarik M, MacGlashan DW, Undem BJ (2005). Nasal sensory nerve populations responding to histamine and capsaicin. J Allergy Clin Immunol.

[B42] Fox H, Lane EB, Whitear M, Spearman RIC and Riley PA (1980). Sensory nerve endings and receptors in fish and amphibians. The Skin of Vertebrates.

